# Bio-Based Vitrimers from 2,5-Furandicarboxylic Acid as Repairable, Reusable, and Recyclable Epoxy Systems

**DOI:** 10.1021/acsapm.2c01774

**Published:** 2022-12-23

**Authors:** Eleonora Manarin, Federico Da Via, Benedetta Rigatelli, Stefano Turri, Gianmarco Griffini

**Affiliations:** Department of Chemistry, Materials and Chemical Engineering “Giulio Natta”, Politecnico di Milano, Piazza Leonardo da Vinci 32, 20133Milano, Italy

**Keywords:** covalent adaptable networks, vitrimers, bio-based, epoxy, DGEBA, FDCA, recycling, repairing

## Abstract

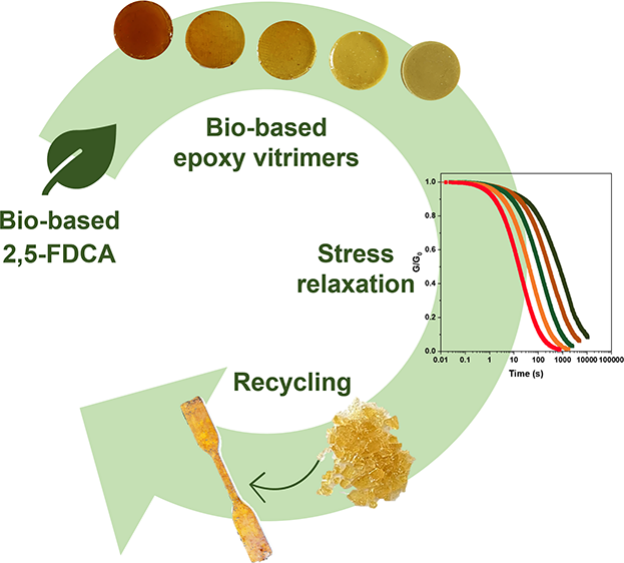

In this work, a series of bio-based epoxy vitrimers were developed by reacting diglycidyl ether of bisphenol A (DGEBA) and bio-based 2,5-furandicarboxylic acid (FDCA) at different molar ratios. Triazabicyclodecene was used as a transesterification catalyst to promote thermally induced exchange reactions. Differential scanning calorimetry, gel content measurements, and Fourier transform infrared spectroscopy were used to study the FDCA-DGEBA crosslinking reaction. The transesterification exchange reaction kinetics of such crosslinked systems was characterized *via* stress relaxation tests, evidencing an Arrhenius-type dependence of the relaxation time on temperature, and an activation energy of the dynamic rearrangement depending on the molar composition. In addition, self-healing, thermoformability, and mechanical recycling were demonstrated for the composition showing the faster topology rearrangement, namely, the FDCA/DGEBA molar ratio equal to 0.6. This work provides the first example of bio-based epoxy vitrimers incorporating FDCA, making these systems of primary importance in the field of reversible, high-performance epoxy materials for future circular economy scenarios.

## 1 Introduction

In the context of structural plastics, thermosetting resins are commonly selected as materials of choice because they can offer adequate mechanical, thermal, and chemical performance as typically required in highly demanding applications. Among the different types of thermosetting polymeric systems, epoxy resins are often preferred for the fabrication of high strength systems due to their high crosslinking density, low shrinkage, high rigidity, low creep, and suitable chemical inertness, thermal stability, and solvent resistance.^1^ However, the thermosetting nature of epoxy systems makes their reprocessing, repairing, or remolding impossible to be undertaken by conventional re-melting processes used for thermoplastics, thus posing serious issues in terms of their useful life span as well as their end of life management.^2^ Within this framework, recent progresses in organic and polymer chemistry offer innovative pathways that may help to improve the life cycle of epoxy systems by predictive material design.^3^ In particular, the incorporation of dynamic crosslinking bonds in their macromolecular network represents a powerful chemical tool to equip these thermosets with functionalities, potentially enabling their repairing, reprocessing, and reuse.^4,5^ To that end, covalent adaptable networks (CANs) provide tremendous opportunities for structural reconfiguration of the material in response to various external stimuli,^3,6,7^ relying on dissociative (e.g., Diels–Alder systems)^8−11^ or associative (e.g., vitrimers)^12,13^ chemistries to achieve reversible covalent bond rearrangement and successive repairing, reshaping, reprocessing, or recycling.^14−16^ In the broad area of covalent associative bond exchange reactions, transesterification processes have been among the most widely explored to prepare thermally reprocessable thermosets.^17−19^ In their seminal work, Leibler and co-workers laid the foundations of the field, reporting on the first epoxy vitrimeric systems based on temperature-induced transesterification exchange reactions in a mixture of polycarboxylic fatty acids and diglycidyl ether of bisphenol A (DGEBA) in the presence of a zinc-based catalyst.^20^

In more recent years, the development of increasingly more performing transesterification vitrimers^21^ has been progressively characterized by the incorporation of bio-based precursors in their formulation as substitutes of (or in conjunction with) petroleum-based ones, in line with the requirements of green chemistry and circular economy.^22−24^ In particular, different bio-based platforms have entered the vitrimer arena as alternative feedstock to achieve more sustainable, reversible material systems, including epoxidized soybean oil,^25,26^ rosin derivatives,^26^ lignin,^27,28^ isosorbide,^29^ eugenol,^30^ and catechol.^31^

In the context of valuable monomers for the production of biopolymeric materials of potential industrial interest, 2,5-furandicarboxylic acid (FDCA) has attracted a great deal of attention in the past decade given the extensive progresses made in its production routes from different biomass sources with high yield and affordable costs.^32−35^ In this respect, FDCA has been traditionally considered as a bio-based drop-in alternative to terephthalic acid in the production of polyester materials (e.g., polyethylene 2,5-furandicarboxylate, PEF) for various applications, given the excellent thermo-mechanical and barrier properties offered by the incorporation of the compact FDCA structure in the macromolecular network.^36−38^ On the contrary, no examples of the use of FDCA in the field of vitrimers based on heat-induced transesterification reactions have appeared in the literature, despite its potential as a high-performance bio-based source of carboxylic acid groups readily available for dynamic bond exchanges.

To bridge this gap, in this work, we developed a series of bio-based vitrimeric systems based on the reaction of FDCA with DGEBA, in the presence of suitable amounts of triazabicyclodecene (TBD) as a transesterification catalyst. The rigid structure of the two monomers was exploited to provide the resulting dynamic epoxy material with high thermal stability and suitable mechanical response for prospective use in high-performance applications. Moreover, the straightforward formulation process, not requiring solvents or additional functionalization steps, makes these materials potentially suitable for eventual large-scale production. To the best of our knowledge, this represents the first report on the use of bio-derived FDCA in thermally repairable, remoldable, and reprocessable vitrimers, making these systems of critical importance in the field of sustainable reversible epoxy materials for future circular economy scenarios.

## 2 Materials and Methods

### 2.1 Materials

DGEBA, TBD, ethanol, and tetrahydrofuran (THF) were purchased from Merck. Bio-based FDCA (see Figure S1 in the Supporting Information for ^1^H-NMR) was obtained from Nanjing Confidence Chemical Co. Ltd. DGEBA was used as received. TBD was used after purification by recrystallization from solvent (ethanol). As received bio-based FDCA (in powder form) was dried in a vacuum oven at 50 °C overnight, then manually crushed in a mortar, and sieved (50 μm) prior to use.

### 2.2 Preparation of Epoxy Vitrimers Based on FDCA and DGEBA

First, DGEBA was poured into a beaker and maintained under magnetic stirring and nitrogen flux at 105 °C for 10 min to allow complete melting. A suitable amount of milled and sieved FDCA powder was then slowly added to the melted DGEBA over the course of 30 min to ensure optimal solid dispersion in the liquid phase, and the suspension was maintained under magnetic stirring at 105 °C for another 15 min to allow homogenization. Subsequently, freshly recrystallized TBD was added to the FDCA-DGEBA system and the resulting three-component formulation was rapidly poured into a pre-heated (150 °C) silicone mold and placed in a vacuum oven at 50 °C for 1 h to allow the removal of residual trapped gas bubbles. After that, the curing process was performed by thermal treatment at 150 °C for 90 min, allowing achievement of complete crosslinking as confirmed by calorimetric and gel content measurements (see the following sections for details).

### 2.3 Characterization Methods

^1^H-NMR (400 MHz) spectra were recorded on a Bruker Avance 400 using deuterated dimethyl sulfoxide (DMSO-*d*_6_) as a solvent.

Fourier transform infrared (FTIR) spectra were collected with a Thermo Scientific NICOLET Nexus SC-74 FTIR spectrometer. The samples for the analysis were prepared by manually grinding the crosslinked material and mixing it with potassium bromide (KBr) powder followed by compression molding of the mixture powder into round windows. Measurements were performed in transmission mode in the 4000–600 cm^–1^ wavenumber range, recording 64 accumulated scans at a resolution of 4 cm^–1^.

Thermogravimetric analyses (TGA) were performed with a TA Instruments TGA Q500. The samples (∼15 mg) were heated from 25 to 800 °C at a heating rate of 20 °C min^–1^. Measurements were performed in an air atmosphere to evaluate the weight loss in standard thermo-oxidative conditions.

Non-isothermal differential scanning calorimetry (DSC) analyses were carried out with a Mettler-Toledo DSC/823e under a nitrogen atmosphere with non-crosslinked samples to assess the temperature and the kinetics of the crosslinking reaction. Uncrosslinked samples were subjected to a thermal cycle from 25 to 250 °C with different heating rates (β = 5, 10, 15, and 20 °C min^–1^) to determine the exothermic peak temperature of the crosslinking reaction (Tp), the latter being related to the crosslinking temperature. The Ozawa and the Kissinger–Akahira–Sunose (KAS) methods^39,40^ were used to calculate the apparent kinetic activation energy of the curing process (*E*_a_) according to the following equations (eqs 1 and 2), respectively:
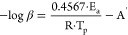
1
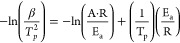
2where *A*′ and *A* are the pre-index factors of the Ozawa and KAS methods, respectively, and *R* is the universal gas constant.

The glass transition temperature (*T*_g_) of the crosslinked materials was determined with a three-run DSC analysis. The sample (5–15 mg) was sealed in an aluminum crucible and heated under a nitrogen atmosphere from 25 to 250 °C, cooled to 25 °C, and then heated again to 250 °C. The first temperature ramp was performed at 10 °C min^–1^, while the second and third ones were performed at 20 °C min^–1^.

The effectiveness of the crosslinking reaction was confirmed *via* gravimetric gel content measurements. The solid sample (*W*_s_ ≈ 1 g) was immersed in 50 mL of THF (good solvent for both DGEBA and FDCA) and left at room temperature under continuous stirring for 24 h. After that time, the solution with the remaining swollen sample was filtered. The solid sample was dried in a vacuum oven at 60 °C for 24 h to completely remove the solvent and weighed until a constant recovered mass (*W*_d_) was recorded. Each sample was tested at least three times to ensure accuracy and reproducibility. The solid extracted fraction (GEL%) was calculated as per eq 3:
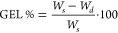
3Stress relaxation experiments were performed with a TA Instruments DHR-2 rheometer with a 25 mm plate-plate geometry at 160, 170, and 180 °C. The sample thickness ranged between 0.8 and 1.5 mm. After a 10 min temperature equilibration step, a 1% torsional strain step was applied, and the relaxation storage shear modulus was monitored as a function of time. Both these steps were performed using a constant normal force of 10 N to ensure good contact between the sample (disk) and the top and bottom plates. Based on the Maxwell’s model for viscoelastic fluids, the relaxation time τ* characteristic of each system at a given temperature was considered equal to the time required at that temperature for the relaxation modulus *G*′(*t*) to reach ∼37% of its starting value *G*′(*0*), namely, *G*′(*t*)*/G*′(*0*) *=* 1/*e*.^41^ Then, an Arrhenius-type law (eq 4) was used to describe the change in τ* as a function of temperature as network relaxations evolve as a consequence of the associative exchange reactions:
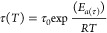
4where τ_0_ is a pre-exponential factor and *E*_a(τ)_ is the activation energy of the exchange reaction.

Dynamic mechanical analyses (DMA) were carried out on crosslinked systems in tensile mode with a Mettler Toledo DMA/SDTA 861e dynamic mechanical analyzer. Rectangular specimens with a thickness of 0.5 mm and a width of 3.5 mm were tested in displacement-controlled oscillation using an amplitude of 10 μm in combination with a frequency of 1 Hz and a clamping distance of 10.5 mm. The loss factors (tan δ) and the storage modulus (*E*′) were monitored over the 25–225 °C temperature range at a heating rate of 2 °C min^–1^. DMA measurements were also used to evaluate the modulus in the rubbery plateau and to quantify the crosslinking density ν (moles of crosslinking sites per unit volume, mol cm^–3^) of each sample according to the rubber elasticity theory (eq 5):
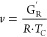
5

where *T*_C_ is the characteristic temperature and *G*_R_^′^ is the shear storage modulus in the rubbery plateau at *T*_C_. *G*_R_^′^ is correlated to the tensile storage modulus in the rubbery plateau *E*_R_^′^ according to eq 6, in which *n* is the Poisson’s ratio (equal to 0.5 for elastically deformed incompressible isotropic materials). *T*_C_ was taken at 30 °C above the *T*_g_ of the sample under test.
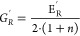
6The topology freezing transition temperature (*T*_v_) of the vitrimeric systems was determined as the point where a viscosity (η) of 10^12^ Pa s is reached, as often reported in the literature.^31,42,43^ According to the Arrhenius relation, the *T*_v_ can thus be calculated from stress–relaxation experiments combining eq 6 and eq 7:
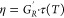
7Scratch healing was evaluated on cured samples by means of optical micrographs recorded through an Olympus BX-60 reflected-light optical microscope equipped with an Infinity 2 digital camera.

## 3 Results and Discussion

### 3.1 Study of the Crosslinking Reaction between FDCA and DGEBA

**Figure 1:**
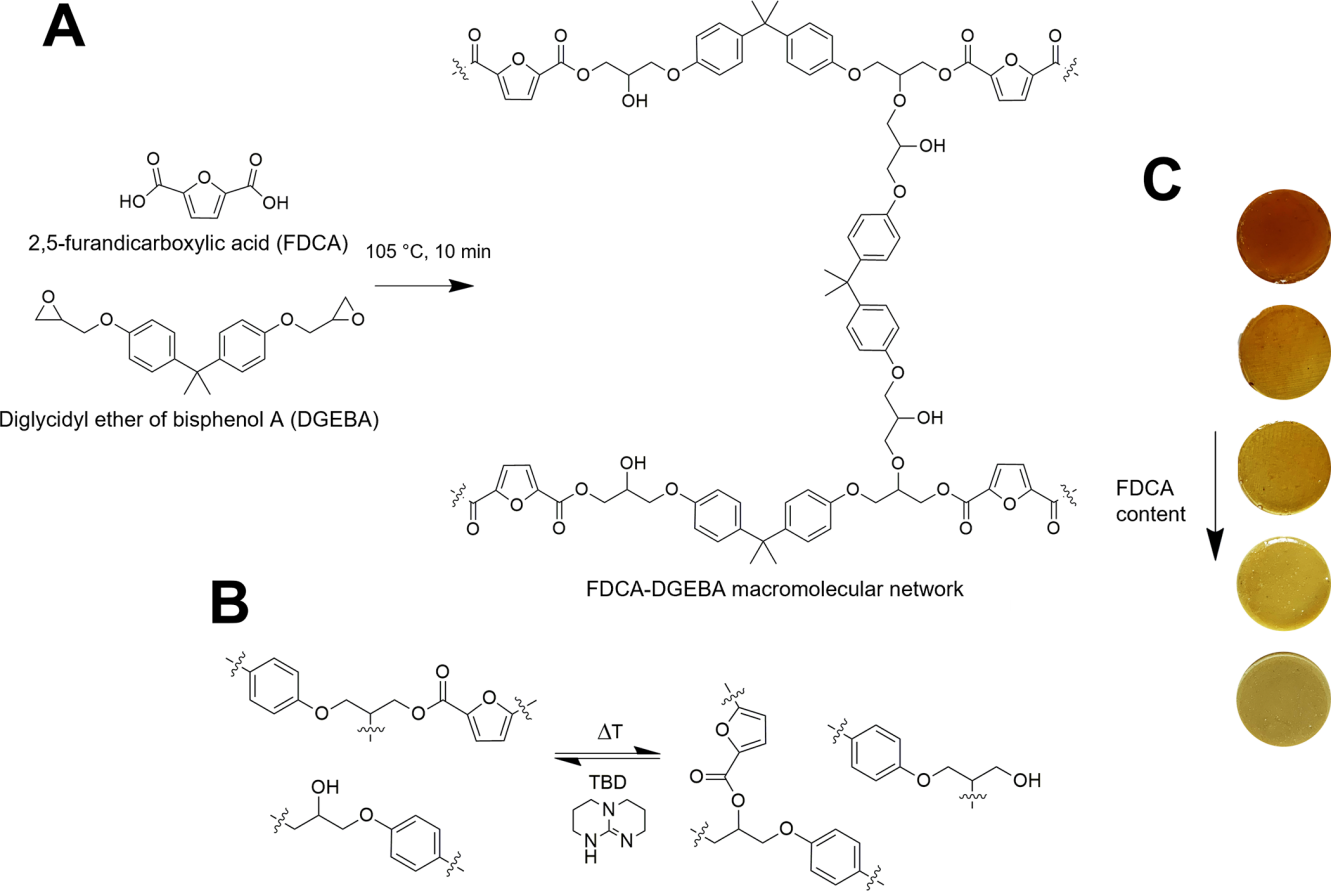
Schematic representation of (A) the synthesized epoxy networks from the FDCA-DGEBA heterophase reaction and (B) catalyst-assisted transesterification reaction. (C) Photographic images of crosslinked FDCA-DGEBA cylindrical samples (20 mm diameter, 5 mm thickness) at increasing FDCA content.

Epoxy vitrimers were prepared *via* a heterophase step-addition reaction between melted epoxy resin DGEBA and finely ground bio-based FDCA powder (Figure 1A). By optimizing the solid-in-liquid dispersion process, a homogeneous transparent solid material with no inclusions could be obtained upon crosslinking, characterized by the typical yellow-brown tint of aromatic epoxy systems,^44,45^ which was found to fade with the increase in FDCA content (Figure 1C).

To study the structure–property relationships of the resulting epoxy networks and their transesterification reaction (Figure 1B), different material compositions were tested, systematically varying the FDCA/DGEBA molar ratios (molar contents for the different formulations are summarized in Table 1). From here on, the resulting materials will be referred to as *x*A/E, with *x* indicating the molar ratio between the acid (A = FDCA) and the epoxy (E = DGEBA) components. A constant catalyst (TBD) load was chosen for all the formulations (5 mol % vs mols of epoxy groups, *viz.* 10 mol % vs mols of DGEBA), in line with a previous report.^46^

**Table 1:** Molar Composition, Reaction Enthalpy (Δ*H*), *E*_a_ of the Crosslinking Process Calculated with the Ozawa and the KAS Methods, and Gravimetric GEL% of the Different Epoxy Vitrimer Formulations

sample^a^	FDCA (mol)	DGEBA (mol)	Δ*H*(J g^–1^)	*E*_a,Ozawa_(kJ mol^–1^)	*E*_a,KAS_(kJ mol^–1^)	GEL%
0.4A/E	4.696 × 10^–3^	1.174 × 10^–2^	69.7 ± 2.98	93.7	91.6	98.3 ± 1.03
0.6A/E	7.044 × 10^–3^	1.174 × 10^–2^	152 ± 5.77	82.4	79.5	98.7 ± 0.57
0.8A/E	9.392 × 10^–3^	1.174 × 10^–2^	190 ± 7.57	81.3	78.4	95.4 ± 1.68
1.0A/E	1.174 × 10^–2^	1.174 × 10^–2^	245 ± 8.22	78.3	75.3	95.0 ± 1.03
1.2A/E	1.409 × 10^–2^	1.174 × 10^–2^	248 ± 5.32	78.3	75.4	90.6 ± 0.90

a Samples are identified based on the molar ratio between FDCA (A) and DGEBA (E).

The curing time and temperature were investigated by preliminary DSC analyses on the non-crosslinked materials.

For each system, measurements were carried out at increasing heating rates (β = 5, 10, 15, and 20 °C min^–1^) to evaluate the temperature of the exothermic peak (*T*_p_) related to the crosslinking reaction. The activation energy (*E*_a_) of the curing process for all formulations was computed using the Ozawa (eq 1) and the KAS (eq 2) methods (the associated linear regression curves are shown in Figure S2 in the Supporting Information).

As shown in Table 1, the reaction enthalpy of the curing process increased with FDCA content as more reactive sites are present.^47^ In accordance, the activation energy of the curing process decreased slightly as the FDCA content increased, likely due to progressively larger availability of carboxylic groups for the epoxy ring opening reaction, thus promoting the reaction process.^48,49^ The comparatively low values of *E*_a_ found in all formulations are in line with those reported for analogous epoxy systems,^50,51^ indicating that this reaction is favored irrespective of the molar composition investigated.

The curing conversion was found to be ∼100% after the curing cycle (i.e., 150 °C for 90 min) for all the formulations, as confirmed from DSC analyses by the complete disappearance of the exothermic peak associated to the curing reaction after the thermal treatment (Figure S3 in the Supporting Information).

Gravimetric gel content measurements were employed to evaluate the extent of crosslinking of the A/E systems in terms of residual soluble and insoluble fractions upon solvent (THF) extraction. The amount of insoluble fraction (GEL%), calculated according to eq 3, was found to slightly decrease with increasing the acid-to-epoxy molar ratio, indicating the formation of a looser crosslinked network for high FDCA content systems. This effect is more evident in the formulation with the largest molar excess of FDCA *vs* DGEBA (i.e., 1.2A/E), where a GEL% ≈ 90% was reported. This behavior may be explained by the lower degree of conversion of the acid-epoxy reaction for higher-acid-group-content formulations,^52^ resulting from the presence of unreacted acid groups from FDCA molecules after the curing cycle. Considering GEL% = 95% as a lower threshold for a completely crosslinked thermoset material,^53^ the 1.2A/E composition was thus excluded from further analysis.

FTIR spectroscopy was used on the different A/E formulations to monitor the oxirane ring opening reaction in DGEBA and confirm successful incorporation in the crosslinked systems (Figure S4 in the Supporting Information). In all systems, no residual signals centered at around 915 cm^–1^ associated with the CH_2_-O-CH bending deformation of the epoxy functional group in DGEBA were found after the curing process, indicating successful opening of epoxy rings.^54,55^ Moreover, when comparing the spectra of the crosslinked materials with those of unreacted FDCA, a shift of the C=O stretching signal toward higher frequencies was observed, from ∼1690 cm^–1^ (carboxylic acid) in FDCA to ∼1730 cm^–1^ (ester group) in the crosslinked systems.^56^ Finally, for increasing FDCA concentrations (going from 0.4A/E to 1.0A/E), this peak was found to increase in intensity as a result of the increase in the concentration of ester groups in the formulation. These pieces of evidence indicate successful reaction between FDCA and DGEBA and formation of a covalently linked structure.

### 3.2 Thermal Characterization of Crosslinked Systems

**Figure 2:**
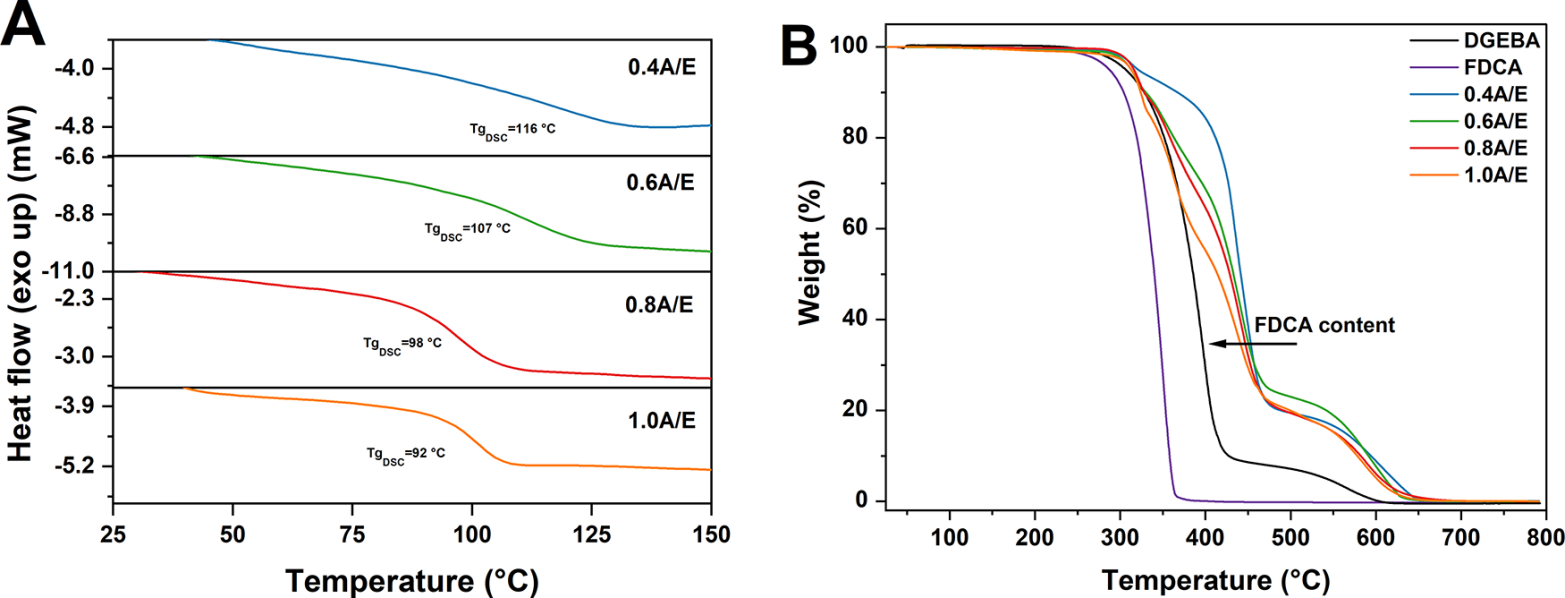
(A) DSC plots and (B) TGA curves of the A/E epoxy systems at varying composition.

The thermal response of the obtained A/E crosslinked systems was assessed by means of non-isothermal DSC analysis and TGA measurements. As shown in Figure 2A, the *T*_g_ of the crosslinked samples as obtained from DSC analysis (denoted as *T*_g,DSC_) was found to be in the 90–120 °C range, in line with the values reported for conventional amine-cured^57^ and anhydride-cured epoxy systems,^58−61^ with a significant dependence on the FDCA-DGEBA relative proportions within the macromolecular network. In particular, an evident decrease in T_g,DSC_ could be observed by increasing (decreasing) the FDCA (DGEBA) content in the formulation. This trend may be correlated with an increasingly higher macromolecular mobility found in systems with a progressively lower concentration of rigid aromatic rings from DGEBA as the FDCA/DGEBA molar ratios increase, in line with GEL% measurements.

The thermo-oxidative stability of the A/E systems was studied by means of TGA measurements in air (Figure 2B, Table 2, and Figure S5 in the Supporting Information). As shown in Figure 2B and Figure S5, no weight losses were observed in the crosslinked materials up to ∼300 °C, indicating their excellent response at potential processing/application temperatures (≤300 °C) irrespective of their chemical composition. The thermo-oxidative stability of the proposed epoxy systems is in line with reported literature data.^62^ In particular, higher thermal stabilities were found *vs* the starting components (namely, FDCA and DGEBA), confirming the successful formation of a robust three-dimensional macromolecular covalent structure during the crosslinking process. For higher temperatures, a two-stage thermal degradation process was found, with a first mass loss event at *T* = 300–400 °C likely associated with the decomposition of the FDCA-based domains and a second mass loss event at *T* > 470 °C related to the complete disruption of the macromolecular network. This behavior is in line with analogous aromatic epoxy systems, also in the presence of similar furan-based precursors.^63−66^ Interestingly, A/E systems with increasing amounts of FDCA were found to exhibit progressively lower thermo-oxidative stability in the 300–470 °C temperature range. These trends are confirmed by the values shown in Table 2 for the degradation temperatures at 2% weight loss (*T*_2%_) and 10% weight loss (*T*_10%_) as well as for the maximum mass loss derivative temperature (*T*_DTGA(max)_). The final char residue at 800 °C (*R*_800_) was found in all cases to be negligible, indicating complete thermo-oxidative degradation of all systems at that temperature. The lower thermal stability of the FDCA rich networks may be associated with the lower gel content found in those formulations (see Table 1) and to the possible presence of some residual, unreacted −COOH groups.

**Table 2:** Characteristic Degradation Temperatures and Final Char Residue for the A/E Systems, as Obtained from TGA Measurements in Air

sample	*T*_2%_ (°C)	*T*_10%_ (°C)	*T*_DTGA(max)_ (°C)	*R*_800_ (%)
FDCA	263	304	355	0.00
DGEBA	283	328	401	0.00
0.4A/E	302	367	435	0.05
0.6A/E	297	330	440	0.03
0.8A/E	303	328	441	0.01
1.0A/E	290	324	363	0.01

### 3.3 Mechanical Characterization of the Crosslinked Systems

**Figure 3:**
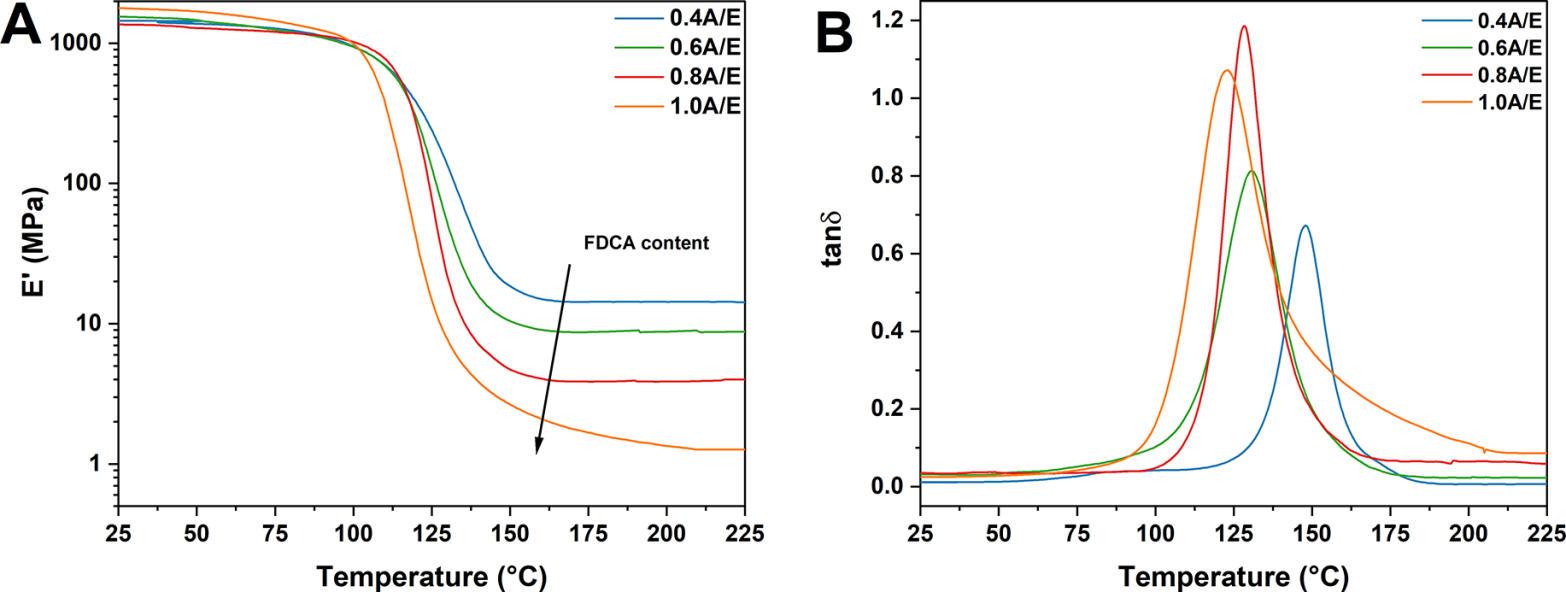
(A) Tensile storage modulus (*E*′) and (B) tan δ curves as a function of temperature for A/E systems, as obtained from tensile-mode DMA measurements in temperature sweep scans.

DMA measurements in tensile mode were employed to study the thermo-mechanical response of the A/E epoxy systems at varying FDCA/DGEBA molar compositions (Figure 3 and Table 3). All crosslinked materials exhibited a room-temperature (25 °C) tensile storage modulus *E*_G_^′^ in the order of 1.5–2 GPa, in line with conventional thermosetting epoxy resins used in commercial applications,^67,68^ with a glassy plateau extending up to 100 °C in all formulations. The glass transition temperature, taken as the peak temperature of the tan δ curve (*T*_g,DMA_, Figure 3B), was found to progressively decrease for increasing FDCA content in the system, in agreement with the trends observed from DSC analyses (the slightly higher values recorded for *T*_g,DMA_*vs T*_g,DSC_ are associated with the different heating rates used, namely, 2 °C min^–1^*vs* 20 °C min^–1^ for DMA and DSC measurements, respectively, and with the kinetic nature of the glass transition process). Accordingly, a similar response was also observed for the storage modulus at the rubbery plateau *E*_R_^′^ (*T* > 140 °C), which showed a decreasing trend as the FDCA/DGEBA molar ratio in the formulation increases. Interestingly, a less defined rubbery plateau and a broader glass transition were found in 1.0A/E compared with the other systems, also reflected in its wider tan δ curve (Figure 3B), likely indicative of a larger heterogeneity in the distribution of chain lengths between crosslinking nodes, leading to an extension of the glass-to-rubber transition to higher temperatures.

**Table 3:** *T*_g_ from DSC and DMA, *E*_G_^′^ at 25 °C, *E*_R_^′^, and ν Obtained from DMA in Tensile Mode

sample	*T*_g,DSC_ (°C)	*T*_g,DMA_ (°C)	*E*_G_^′^ (25 °C) (GPa)	*E*_R_^′^ (MPa)	ν (mol cm^–3^)
0.4A/E	116	147	1.47	15.5	13.7 × 10^–4^
0.6A/E	107	131	1.52	8.76	8.18 × 10^–4^
0.8A/E	98	129	1.36	3.99	3.54 × 10^–4^
1.0A/E	92	123	1.77	1.59	1.11 × 10^–4^

DMA measurements were also used to evaluate the crosslinking density ν of the obtained materials, based on eqs 5 and 6. As reported in Table 3, ν values between ∼14 × 10^–4^ and ∼1 × 10^–4^ mol cm^–3^ were found, generally higher with respect to the values found for an analogous literature-based epoxy system,^61,68^ consistently decreasing for higher FDCA contents, as expected. Interestingly, such values of ν were maintained constant even at higher temperatures, thus confirming the thermosetting nature of these systems and anticipating the associative bond-exchange origin of their dynamic properties, as will be discussed in the following sections.^69^

The mechanical properties of such epoxy vitrimeric systems were tested in tensile mode on a universal testing machine (Figure S6 in the Supporting Information). All materials exhibited a typical rigid response with the tensile elastic modulus (*E*_t_) found to be in the order of ≈2–2.5 GPa, irrespective of the composition. Such values are compatible with literature-based traditional and vitrimeric epoxy systems for high-performance applications (i.e., composite materials, aerospace, and construction).^60,70^

### 3.4 Characterization of the Vitrimeric Behavior

**Figure 4:**
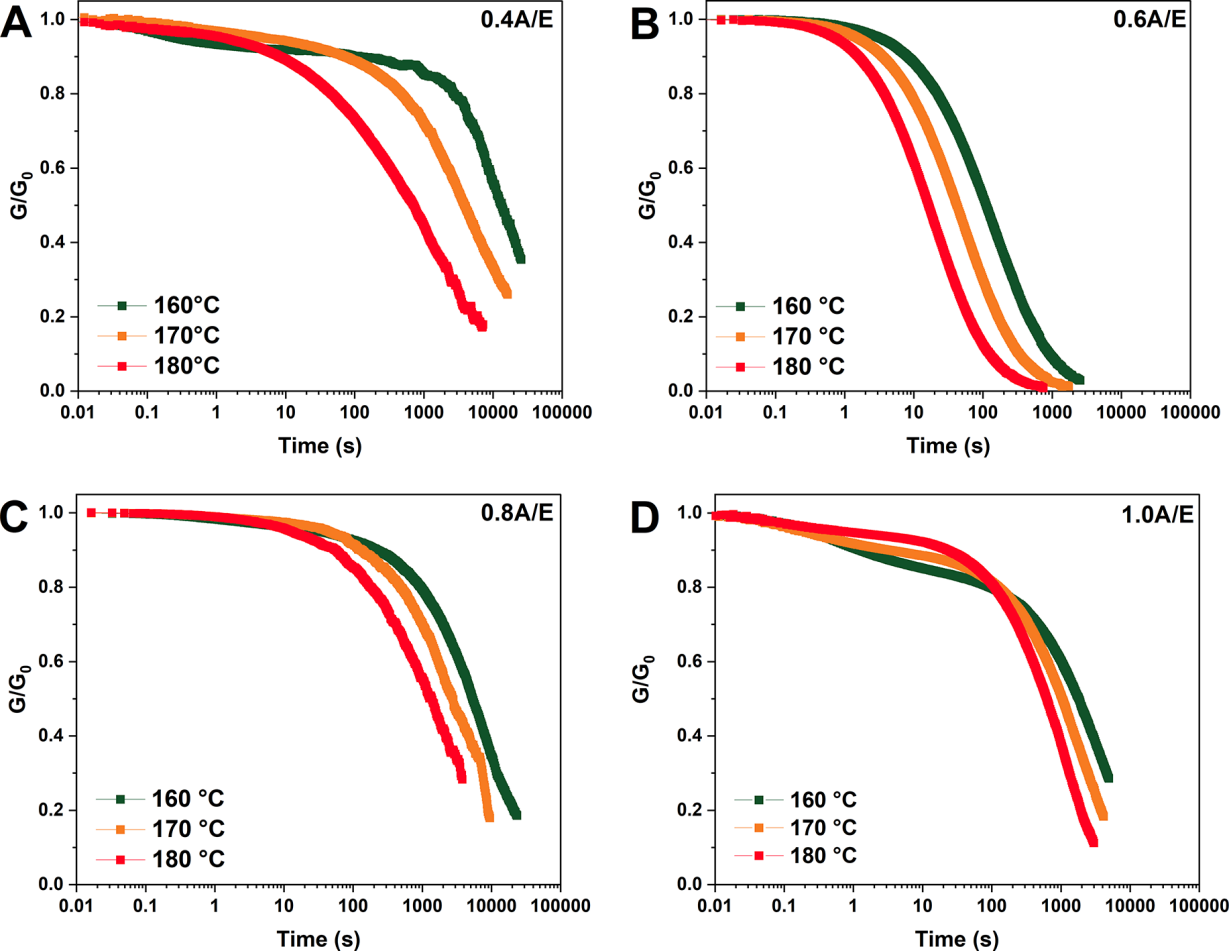
Normalized stress relaxation curves for (A) 0.4A/E, (B) 0.6A/E, (C) 0.8A/E, and (D) 1.0A/E at 160 °C (green), 170 °C (orange), and 180 °C (red).

**Figure 5:**
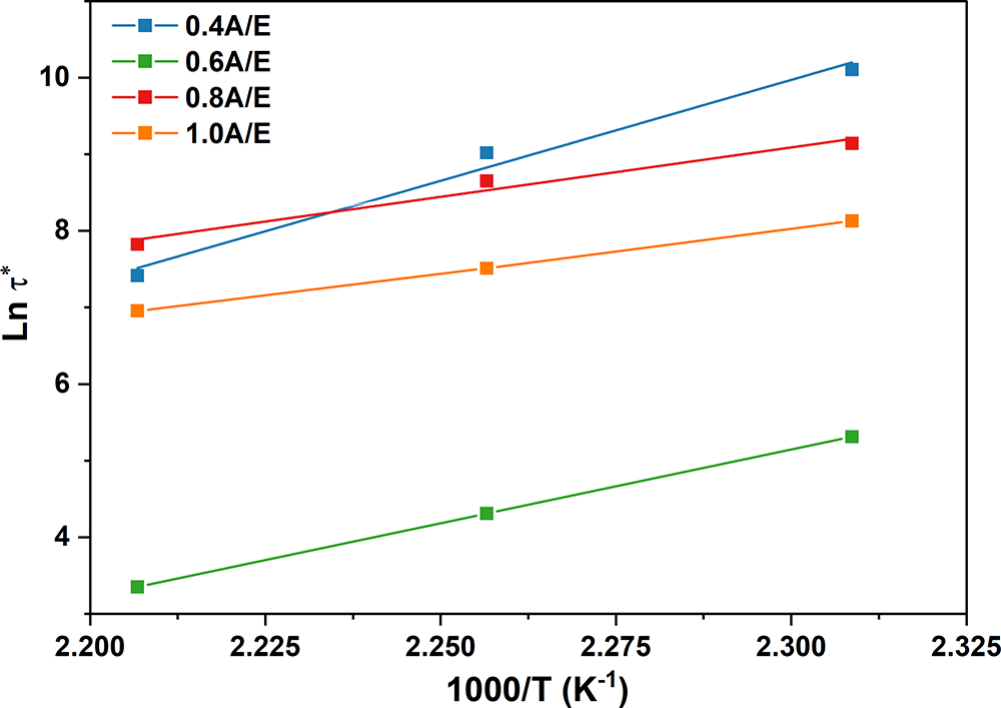
Ln τ* *vs* 1/*T* plot from Arrhenius’ dependence of τ* on temperature. The activation energy *E*_a(τ)_ of the dynamic exchange reaction is calculated as the slope of the curve.

The dynamic properties of the A/E epoxy systems were analyzed by means of stress relaxation experiments, monitoring the response of the materials in terms of relaxation modulus *G*′(*t*) over time at different temperatures, namely, 160, 170, and 180 °C. As shown in Figure 4, where *G*′(*t*) normalized with respect to the corresponding *G*′(*0*) value recorded at time *t* = 0 s is plotted for all A/E systems investigated, each formulation displayed evident stress relaxation at temperatures above *T*_g_. The observed trends are indicative of a typical viscoelastic fluid behavior, thus suggesting the presence of exchangeable linkages within the macromolecular network.^43^ To quantify the extent of such behavior, the Maxwell’s model for viscoelastic fluids was employed, estimating the characteristic relaxation time τ* of each A/E system at a given temperature as the time required to achieve *G*′(*t*)/*G*′(*0*) = 1/*e* (≈0.37). Based on the relaxation curves reported in Figure 4, τ* values ranging between 7 h (0.4A/E, 160 °C) and 30 s (0.6A/E, 180 °C) were found, with a marked dependence on the FDCA/DGEBA molar ratio as well as on temperature, being the relaxation processes in vitrimer networks mainly governed by the continuous temperature-dependent bond-exchange reaction kinetics.^69^ In particular, the largest τ* values were observed in the 0.4A/E system (largest excess of epoxy component and therefore higher concentration of nonreversible, ether-like linkages), and for all formulations, they were shown to decrease by increasing the test temperature (Table 4). To further investigate the kinetics of the relaxation processes in the macromolecular networks in such materials *via* associative exchange reactions, an Arrhenius law dependence of τ* on temperature (eq 4) was used. In particular, the activation energy of the dynamic transesterification exchange reactions (*E*_a(τ)_) was calculated as the slope of the curve in a semi-logarithmic plot of τ* as a function of the inverse of the test temperature. As shown in Figure 5, *E*_a(τ)_ was found to decrease for increasing FDCA/DGEBA molar ratio, moving from 219 kJ mol^–1^ in 0.4A/E to 93 kJ mol^–1^ in 1.0A/E. This trend can be explained by considering that an increase in the amount of FDCA in the formulation yields an increased density of active functional groups potentially available for the transesterification exchange reactions, thus making this process less energetically demanding.^71^ However, it should also be emphasized that a change in FDCA concentration at a constant catalyst load (5 mol % vs mols of epoxy groups) in the formulation will lead to a change in the molar ratio between active functional groups and the transesterification catalyst (TBD). In particular, as the amount of FDCA increases, this ratio will decrease, thus progressively slowing down the bond exchange reaction kinetics and ultimately leading to an increase in τ* at a given temperature. Based on these considerations, 0.6A/E appears to exhibit the most appropriate balance between the amount of available active functional groups for the transesterification reaction (in relation to the energy required for the exchange reaction to occur) and catalyst abundance (in relation to the kinetics of the relaxation process). Indeed, such formulation was characterized by an *E*_a(τ)_ of ∼150 kJ mol^–1^, in line with typical values for epoxy vitrimers,^69,72^ but short relaxation times ranging between 3 min at 160 °C and 30 s at 180 °C (Figure S8 in the Supporting Information for additional temperatures tested). Interestingly, such τ* values appear very competitive when compared with those reported for reference epoxy vitrimers recently presented in the literature,^73,74^ making our system particularly appealing for prospective applications.

**Table 4:** *E*_a(τ)_, *T*_v_, and τ* at 160, 170, and 180 °C for the A/E Epoxy Systems

sample	*E*_a(τ)_(kJ mol^–1^)	*T*_v_ (°C)	τ*_160°C_ (s)	τ*_170°C_ (s)	τ*_180°C_ (s)
0.4A/E	219.0	150	24,427	8254	1661
0.6A/E	151.5	94.5	203	74	30
0.8A/E	107.2	105	9327	5704	2499
1.0A/E	93.02	74.5	3389	1829	1049

As expected, the calculated theoretical *T*_v_ (eq 7) of the proposed systems was found to be highly dependent on the formulation. The 0.4A/E system showed a *T*_v_ > *T*_g,DMA_ related to its high *E*_a(τ)_. For this specific material, the viscoelastic liquid flow could only be exploited above 150 °C. For all other systems exhibiting a much lower value of *E*_a(τ)_, *T*_v_ was found to be lower than *T*_g,DMA_. In these cases, once the temperature reaches values higher than *T*_g_, the exchange reactions are rapidly activated, leading to the topological rearrangement of the network.

### 3.5 Material Repairing, Reuse, and Recycling

**Figure 6:**
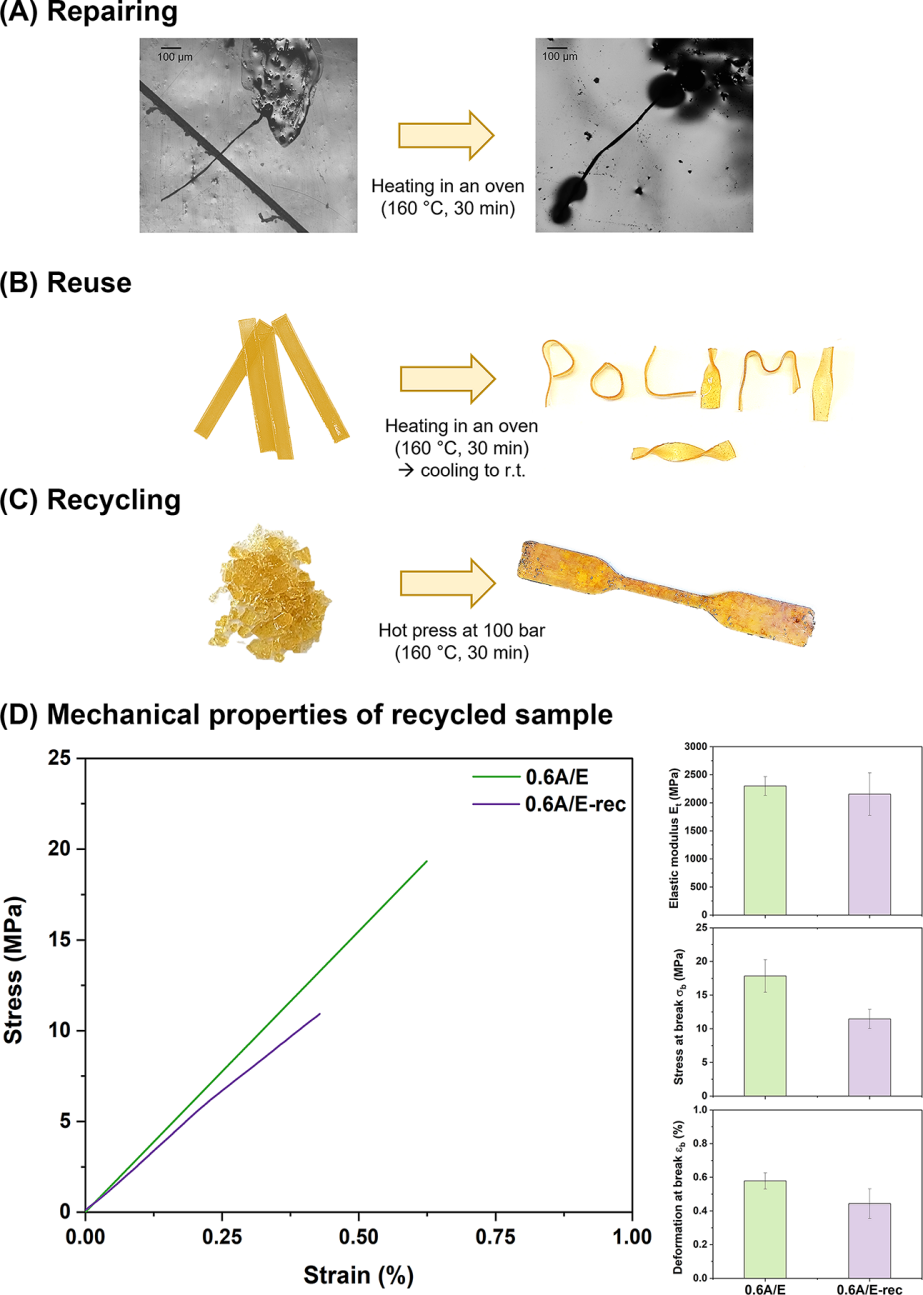
Demonstration of (A) repairing ability *via* thermally induced healing of surface scratches; (B) reuse ability *via* thermoforming reprocessing; (C) mechanical recycling *via* hot-pressing at 100 bar. In all cases, the 0.6A/E system was maintained at 160 °C for 30 min followed by cooling to room temperature (r.t.). (D) Stress–strain curves of pristine (0.6A/E) and mechanically recycled (0.6A/E-rec) FDCA/DGEBA vitrimers obtained from tensile tests, together with histograms reporting elastic modulus (*E*_t_), stress at break (σ_b_), and deformation at break (ε_b_) (standard deviations out of at least five specimens).

Building upon the vitrimeric characteristics of the FDCA/DGEBA systems, their dynamic features were further investigated by assessing the ability of such bio-based epoxy materials to be repaired, reused, and recycled upon suitable thermo-mechanical stimulus (Figure 6). As anticipated, the 0.6A/E formulation was selected as a representative platform, given its favorable characteristics in terms of activation energy for the transesterification reaction and of relaxation times. Encouragingly, the time required for this system to macroscopically exhibit its vitrimeric behavior at a given temperature proved to be aligned with the typical values of τ* extrapolated *via* stress relaxation tests.

The repairing ability of the bio-based epoxy vitrimer was demonstrated through scratch healing tests. In particular, a surface cut of ∼60 μm width and ∼150 μm depth was mechanically induced at room temperature by means of a lancet on a bulk specimen. The damaged material was then heated in a ventilated oven for 30 min at 160 °C. As shown in Figure 6A, upon thermal treatment, the material was able to completely recover its surface features, fully mending the scratch, as a result of the thermally induced stress relaxation and the topological rearrangement of its macromolecular network. Moreover, as expected, the healing kinetics (thus, the healing efficiency at a given thermal treatment condition) is highly dependent on the system analyzed as it is based on chain relaxation resulting from the macromolecular vitrimeric flow (see Figure S7 in the Supporting Information for the corresponding optical micrographs). The healing mechanism can be associated to the transesterification reaction between hydroxyl groups and ester groups at high temperatures, leading to scratch healing without the need of external healing agents.^26,75,76^ In fact, the composition showing the faster topology rearrangement (namely, 0.6A/E) is the only one able to achieve complete healing for the selected time and temperature for the healing cycle. The other systems show partial healing due to the slower relaxation kinetics, thus ultimately requiring a longer time exposure to allow complete scratch recovery.

The possibility to reuse the vitrimer epoxy material was demonstrated in terms of remolding ability *via* thermoforming. To that end, flat panels of pristine 0.6A/E rectangular specimens were produced (75 mm × 3 mm × 8 mm) and maintained at 160 °C for 30 min. During the heating step, such panels were manually formed into various shapes using twizzers and then allowed to cool down to room temperature. As shown in Figure 6B, after the cooling step, a permanent fixed shape could be obtained, confirming that the material underwent complete stress relaxation as a result of its vitrimeric characteristics.

Finally, mechanical recycling was also demonstrated through hot pressing. A pristine 0.6A/E specimen was cryogenically ground into granules and loaded into a dog-bone-shaped steel mold for compression molding (coating with polytetrafluoroethylene was required to prevent adhesion to the mold). A pressure of 100 bar was applied for 30 min at 160 °C followed by cooling to room temperature. As evident from Figure 6C, after the compression molding reprocessing step, the material could be recovered as a singular piece with well-defined shape and no macroscopic defects, as a result of successful granule sintering and favorable thermo-mechanically induced vitrimeric material viscous flow.

To assess the mechanical response of the recycled material, the hot-pressed reprocessed specimens were tested via both DMA in tensile mode and tensile measurements and compared with the as-prepared counterpart. The mechanically recycled samples were shown to exhibit comparable mechanical response to the pristine counterpart (Figure 6D), maintaining the elastic modulus *E*_t_ in the order of 2 GPa; the stress at break (σ_b_) and deformation at break (ε_b_) were found to decrease slightly with respect to the pristine samples, accompanied by a slightly higher standard deviation. This evidence may be related to the presence of voids and defects resulting from the hot-pressing sintering process and to the possible thermal aging of the epoxy system during high-temperature and high-pressure processing. This response was previously observed also on other similar systems.^77^ Moreover, the reprocessed vitrimer exhibited the same dynamic-mechanical characteristics as the pristine one, suggesting that the (less energetically stable) reversible ester bonds break preferentially during the compression molding step, ultimately yielding topological reorganization of the macromolecular network during the macroscopic granule sintering process (see Figure S9 in the Supporting Information for DMA curves of recycled vs pristine materials).

## 4 Conclusions

In this work, bio-based epoxy vitrimers with repairing, reuse, and recycling capabilities were demonstrated. Such bio-based systems were obtained by a heterophase ring opening reaction between melted epoxy resin DGEBA and finely ground bio-based FDCA powder in the presence of suitable amounts of TBD as a transesterification catalyst. Different formulations were investigated by systematically varying the FDCA/DGEBA molar ratio, enabling a detailed investigation of the resulting structure–property relations of the obtained materials. Non-isothermal DSC analysis was employed to determine the activation energy of the crosslinking process through Ozawa and KAS methods, which was found to lie in the typical ranges (75–95 kJ mol^–1^) observed for conventional epoxy systems. The transesterification exchange reaction process was studied by means of rheological stress relaxation tests, showing an Arrhenius-type dependence of the characteristic relaxation times, determined according to the Maxwell’s model for viscoelastic fluids, of the developed materials on temperature, with activation energies varying with the molar composition. The formulation based on a FDCA/DGEBA molar ratio of 0.6 was found to exhibit especially favorable stress relaxation response, with characteristic relaxation times ranging between 3 min at 160 °C and 30 s at 180 °C, making this system particularly interesting when compared with analogous epoxy vitrimers recently proposed in the literature.

The stress relaxation dynamic features of such FDCA/DGEBA vitrimeric systems enabled their straightforward repairing, reuse, and recycling upon mild thermo-mechanical treatment (160 °C, 30 min), thus successfully demonstrating the beneficial effect of thermally induced stress relaxation and topological rearrangement of their macromolecular network on their processability and macroscopic characteristics.

This work provides the first demonstration of bio-based epoxy vitrimers incorporating FDCA and opens pathways in the predictive design of high-performance, sustainable polymeric and composite materials for future application in the context of virtuous circular economy scenarios.

## Supporting Information Available

^1^H-NMR spectra of FDCA; Ozawa and KAS linear regression curves for curing kinetics studies; DSC plots for conversion studies; FTIR spectra of the epoxy vitrimers; DTGA curves of the epoxy vitrimers; mechanical and self-healing properties of the epoxy vitrimers; stress relaxation for 0.6A/E; and DMA of 0.6A/E (pristine and recycled) (PDF)
