# Supplementary material for: Bio-Based Vitrimers from 2,5-Furandicarboxylic Acid as Repairable, Reusable, and Recyclable Epoxy Systems
Source: ACS Appl Polym Mater. 2022 Dec 23;5(1):828–38. doi: 10.1021/acsapm.2c01774 (PMC9841517; doi:10.1021/acsapm.2c01774)
Supplement: Supplementary file 1 — ap2c01774_si_001.pdf [file ap2c01774_si_001.pdf]

Supporting Information

Bio-Based Vitrimers from 2,5-Furandicarboxylic Acid as Repairable, Reusable and Recyclable Epoxy Systems

Eleonora Manarin[a], Federico Da Via[a], Benedetta Rigatelli[a], Stefano Turri[a], Gianmarco Griffini[a*]*

[a]Department of Chemistry, Materials and Chemical Engineering "Giulio Natta", Politecnico di Milano, Piazza Leonardo da Vinci 32, 20133 Milano, (Italy).

[*]Corresponding author: gianmarco.griffini@polimi.it; stefano.turri@polimi.it.

Table of content

1. ^{1}H-NMR spectra of FDCA in DMSO-d_6……………………………………………....................................S3

2. Ozawa and KAS linear regression curves……………………………………………................................. S4

3. DSC plots for uncured and cured epoxy vitrimers…………………………………………………………….S5

4. FTIR spectra of the epoxy vitrimers…………………………………………..S6

5. DTGA curves of the epoxy vitrimers…………………………………………………………………………S7

6. Mechanical properties of the epoxy vitrimers……………………………………………………………...S8

7. Self-healing properties of the epoxy vitrimers…………………………………………………………..S9

8. Rheological stress relaxation curves for 0.6A/E ………………………………..S10

9. DMA curves of pristine and recycled 0.6A/E systems………………………………………...……..... S11

1. ^{1}H-NMR spectra of FDCA

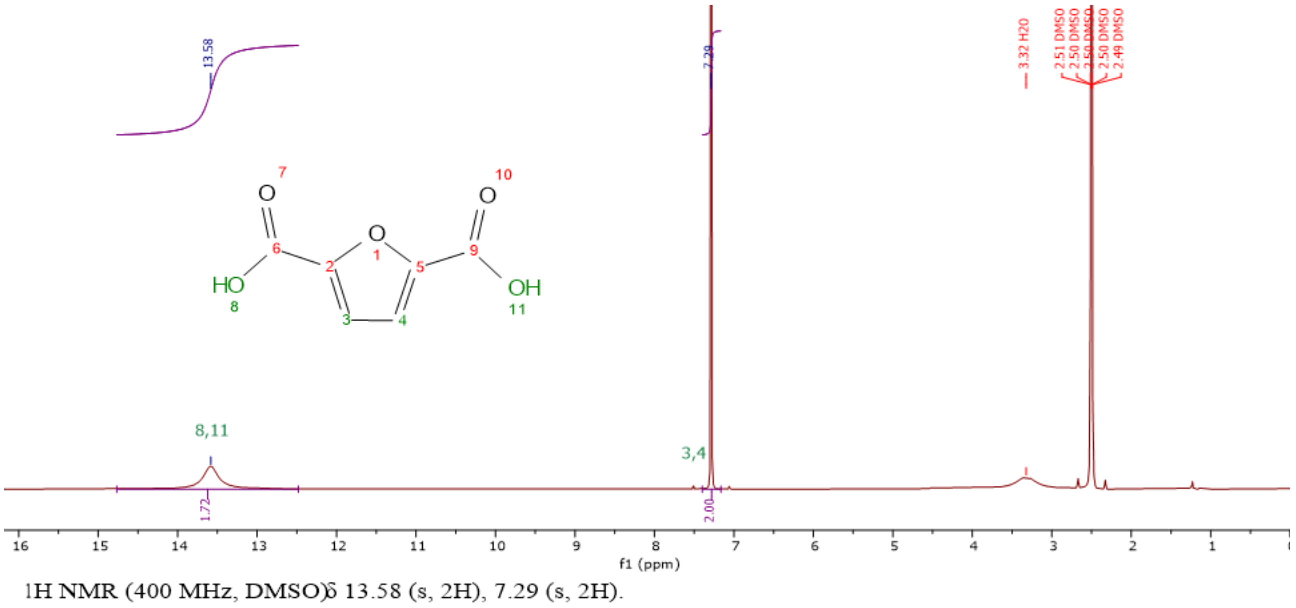

Figure S1. ^{1}H-NMR spectra of bio-based FDCA in DMSO-d_6.

2. Ozawa and KAS linear regression curves

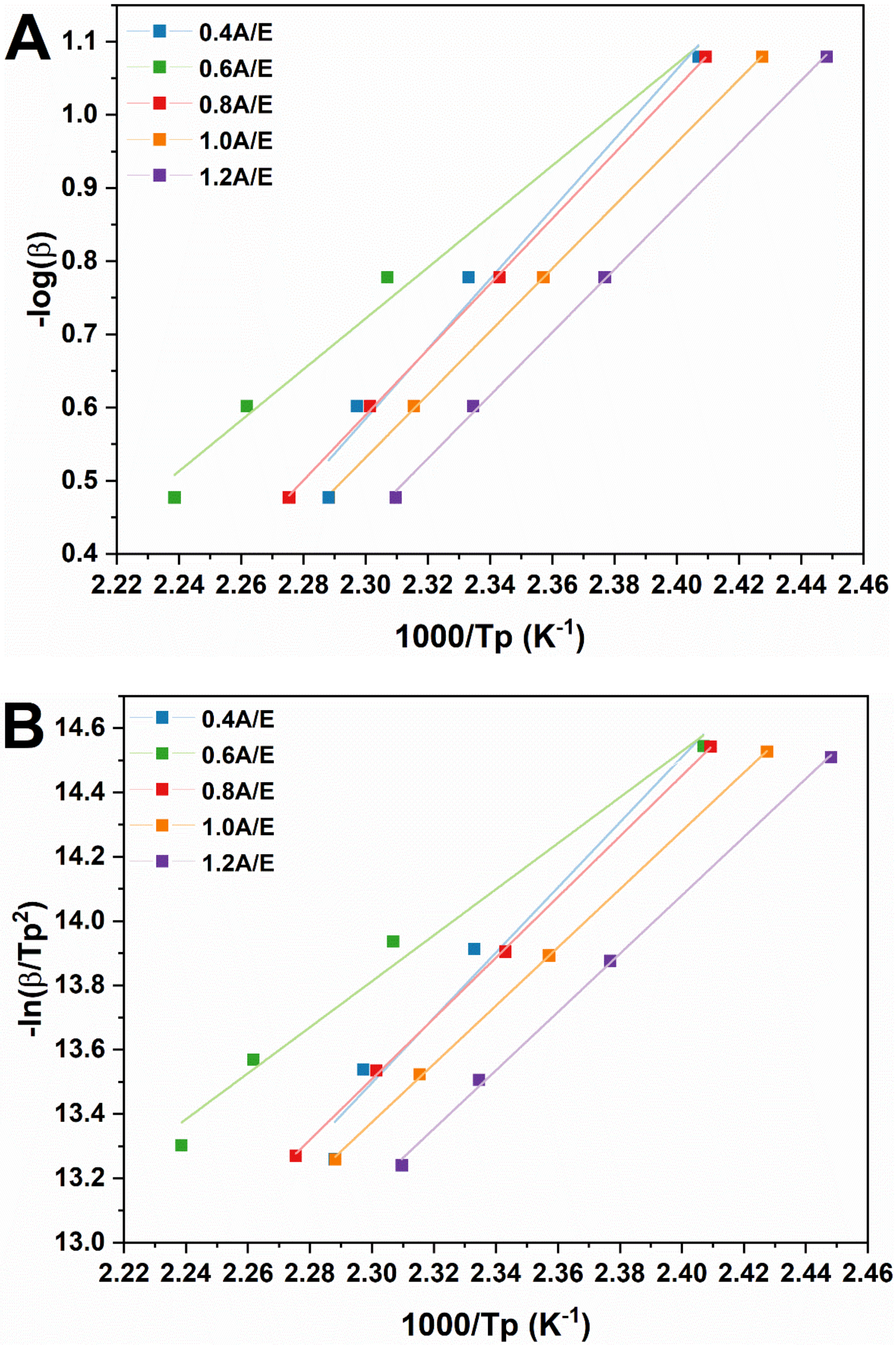

Figure S2. Linear regression curves based on the (A) Ozawa and (B) KAS methods for evaluation of the activation energy of the crosslinking reaction between FDCA and DGEBA for the different formulations investigated.

3. DSC plots for uncured and cured epoxy vitrimers

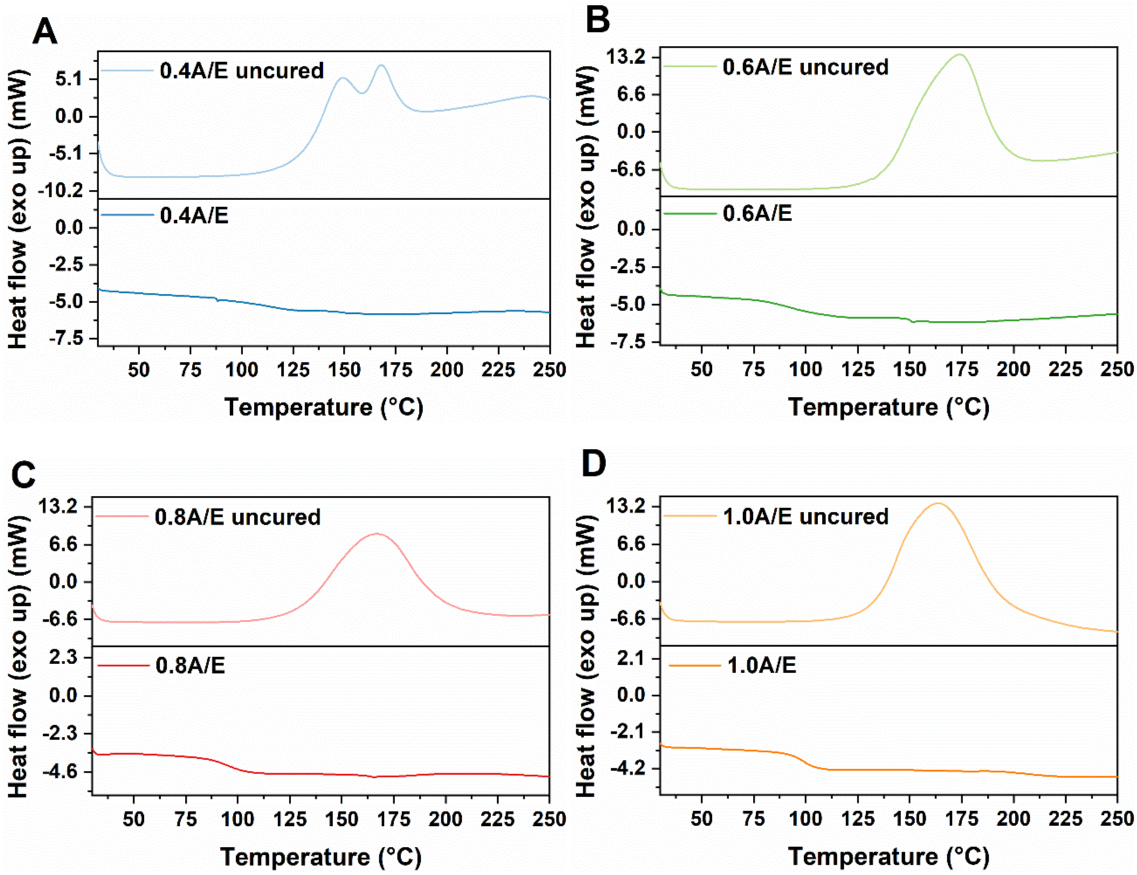

Figure S3. DSC plots for uncured (0% curing conversion) and after curing at 150 °C for 90 min (100% curing conversion) for (A) 0.4A/E, (B) 0.6A/E, (C) 0.8A/E, (D) 1.0A/E.

4. FTIR spectra of the epoxy vitrimers

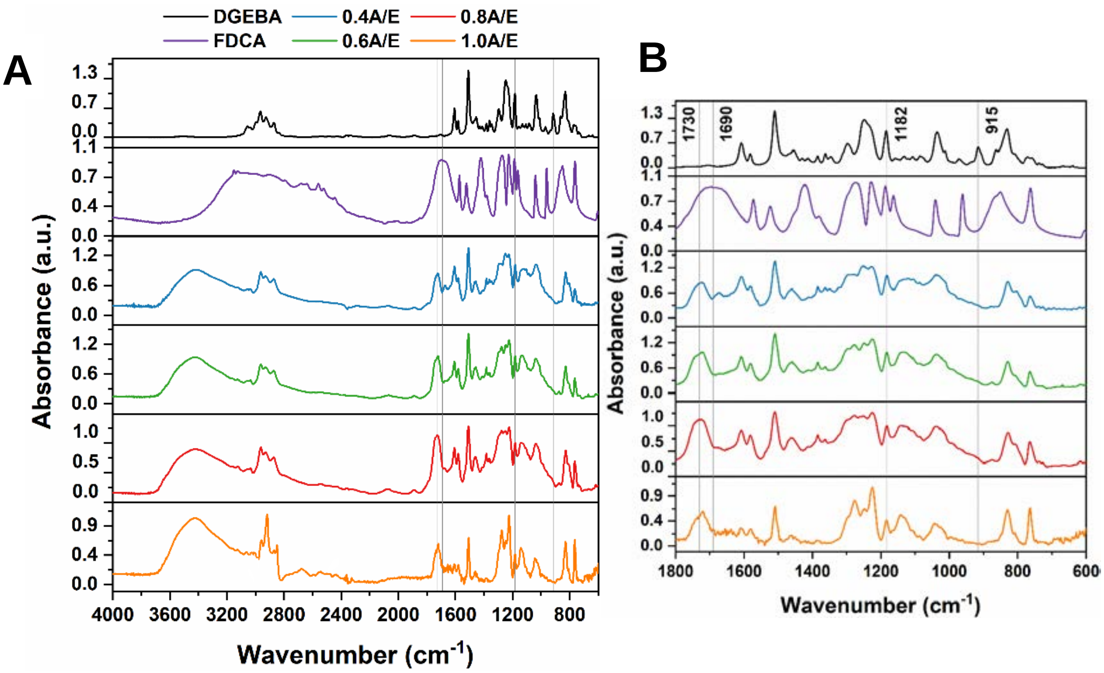

Figure S4. (A) FTIR spectra of the cured FDCA/DGEBA crosslinked systems at varying molar compositions, in comparison with the FTIR spectra of uncured DGEBA and pristine powdered FDCA. (B) Zoomed region (1800-600 cm^{-1}) of the FTIR spectra. All spectra were normalized to the peak centred at 1182 cm^{-1} representing the C-O stretching of the aromatic ring in DGEBA.

5. DTGA curves of the epoxy vitrimers

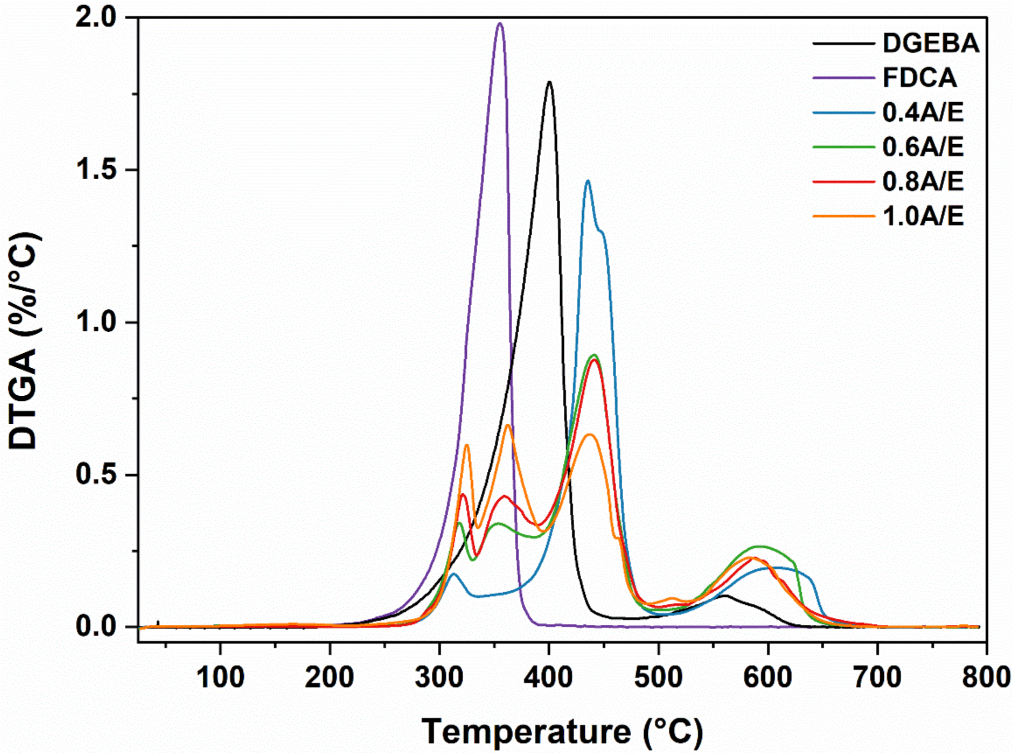

Figure S5. DTGA curves in air flow of A/E epoxy systems at varying composition.

6. Mechanical properties of the epoxy vitrimers

Tensile tests were conducted at 23 °C according to standard DIN EN ISO 527-2 (ASTM D638-14) on a universal testing machine using a Zwick/Roell BT-FR010TH.A50 dynamometer equipped with a 10 kN load cell, applying a displacement rate of 2 mm/min. Deformation was measured using a long-stroke extensometer. Dog-bone shaped (ISO 572-2 type 1B) samples with a gauge length of 27 mm, 10 mm width, and thickness of 2 mm were used. From the resulting stress-strain diagrams, the tensile elastic modulus (E_t) was determined from the slope of the curve between 0.05 % and 0.25 % of the total strain. The ultimate tensile strength (σ_b) and the deformation at break (ε_b) were also evaluated. A minimum of five samples were tested for each formulation.

The resulting stress-strain diagram and the histograms reporting the three parameters of interest evaluated (average value with the corresponding standard deviations) are reported in Figure S6.

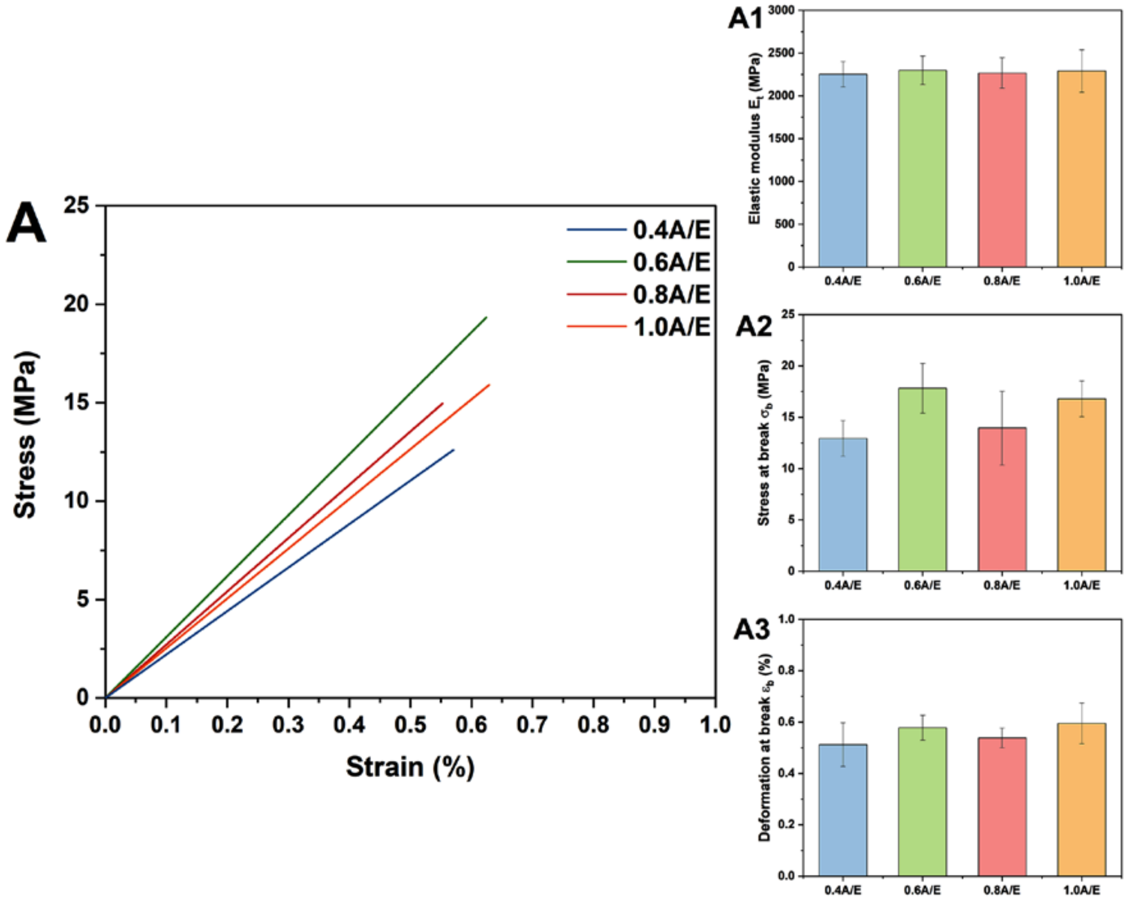

Figure S6. (A) Stress-strain curves for A/E systems obtained from tensile tests. Histograms reporting (A1) elastic modulus (E_t), (A2) stress at break (σ_b) and (A3) deformation at break (ε_b), with the corresponding standard deviations out of at least five specimens.

7. Self-healing properties of the epoxy vitrimers

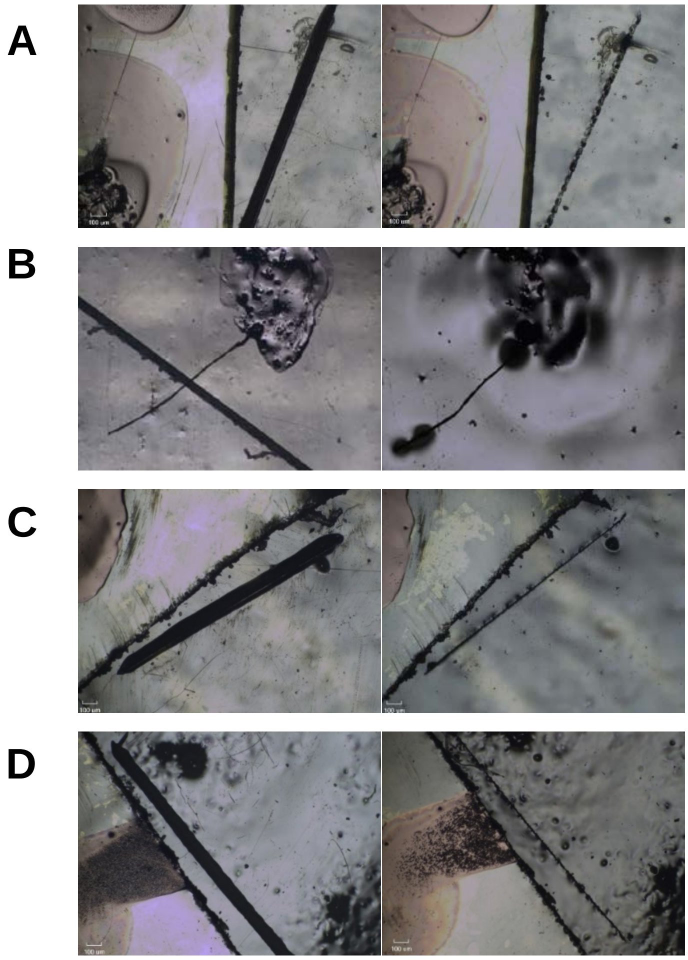

Figure S7. Optical micrographs for (A) 0.4A/E, (B) 0.6A/E, (C) 0.8A/E, (D) 1.0A/E before (left) and after (right) the healing treatment at 160 °C for 30 min, followed by cooling to room temperature (r.t.).

8. Rheological stress relaxation curves for 0.6A/E

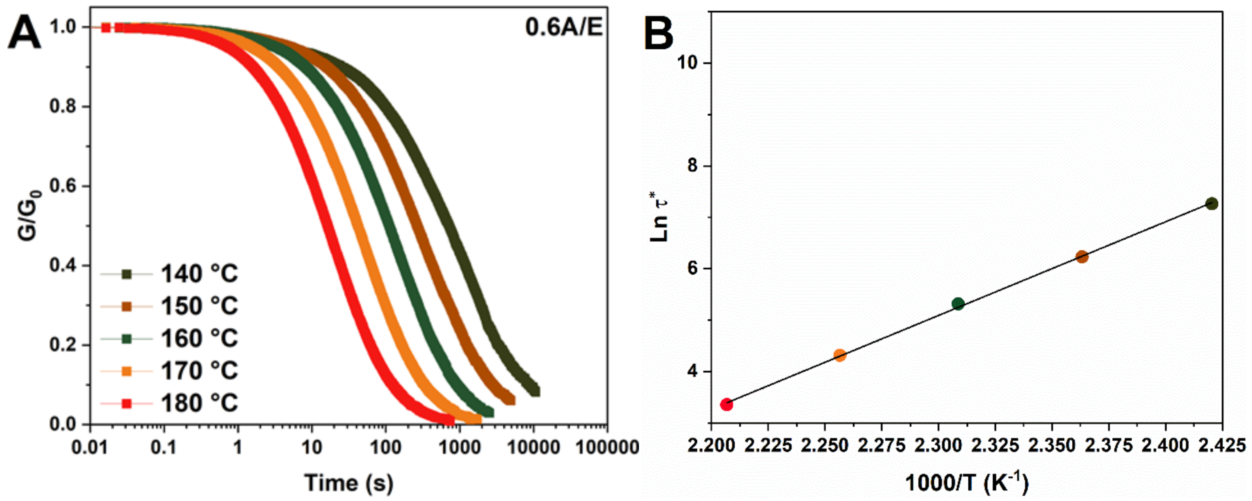

Figure S8. (A) Normalized stress relaxation curves at 140 °C (dark green), 150 °C (brown), 160 °C (green), 170 °C (orange) and 180 °C (red) and (B) Ln τ* vs 1/T Arrhenius plot for 0.6A/E formulation.

9. DMA curves of pristine and recycled 0.6A/E systems

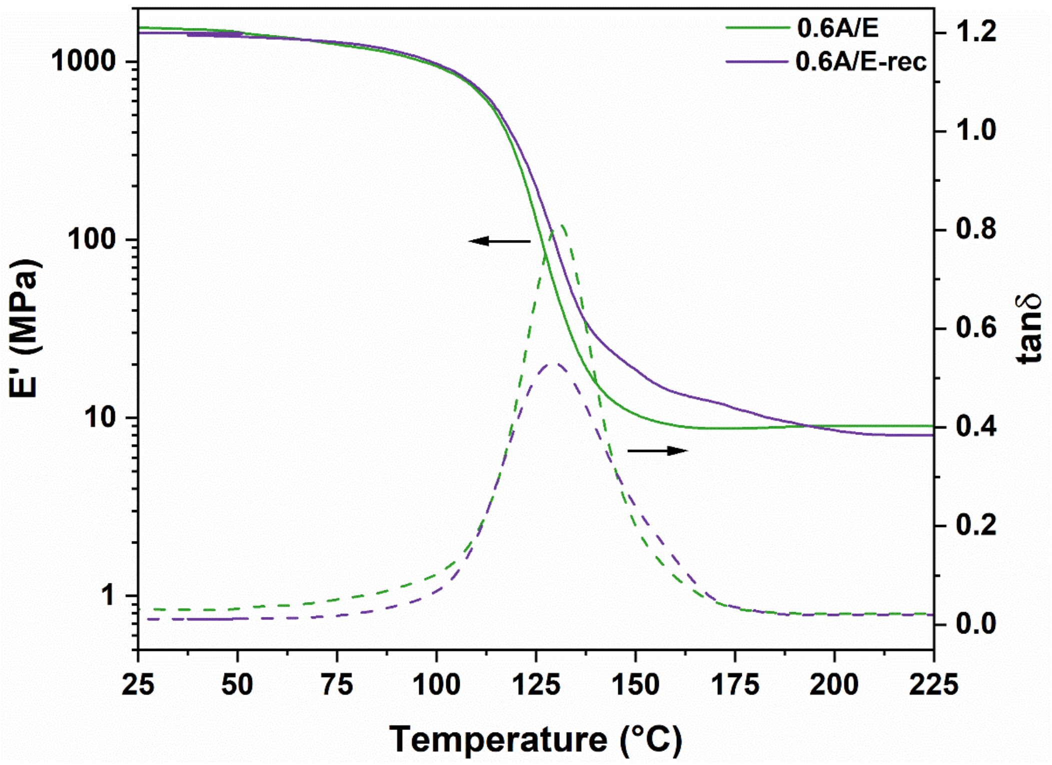

Figure S9. Tensile storage modulus (E′) and tan(δ) curves as a function of temperature obtained from DMA measurements in tensile mode and temperature sweep configuration of pristine (0.6A/E) and mechanically recycled (0.6A/E-rec) FDCA-DGEBA vitrimers.
